# Necessity of Natural Sleep

**Published:** 1891-05

**Authors:** 


### NECESSITY OF NATURAL SLEEP.

Dr. Talcott, Medical Superintendent of the Middletown (N. Y.) Insane Asylum, in a late report, makes some valuable suggestions upon the subject of sleep and sleeplessness. Neither tongue nor pen can too emphatically warn against the dangers which arise from loss of sleep. "If the goddess of sleep fails to respond when we appeal to her for tender and soothing caresses," writes the Doctor, "then, indeed, we are not only harassed in heart, but broken in brain, and made bankrupt in body and mind." The choroid plexuses are the delicate fringings of blood vessels which project into the brain. At night and under favorable conditions they swell and guard the brain from all disturbances ; but when these sentinels of the brain are enfeebled by too great toil, physical disease or mental weariness, they fail to do their work and the antagonist of sleep enters. In the brains of patients who have died insane, there has been found marked disease of these vessels.

One of the most efficient treatments for sleeplessness is massage. This treatment is described in full in the Superintendent's report. The free and indiscriminate use of narcotics is mentioned as one of the most frequent causes of insanity. They are principally used by the overworked, the worrying and discontented. Carlyle says, " The race of life has become intense ; the runners are treading on each other's heels ; woe to him who stops to tie his shoe strings." Dr. Talcott suggests a remedy in these words : " National decay can be averted only by a general reform in our methods of living, and foremost in the line of reform rises a grim and persistent demand for necessary and recuperative sleep."

